# VEGF may contribute to macrophage recruitment and M2 polarization in the decidua

**DOI:** 10.1371/journal.pone.0191040

**Published:** 2018-01-11

**Authors:** Karen C. Wheeler, Manoj K. Jena, Bhola S. Pradhan, Neha Nayak, Subhendu Das, Chaur-Dong Hsu, David S. Wheeler, Kang Chen, Nihar R. Nayak

**Affiliations:** 1 Department of Obstetrics and Gynecology, Wayne State University, Detroit, Michigan, United States of America; 2 Department of Biotechnology, School of Bioengineering and Biosciences, Lovely Professional University (LPU), Phagwara, Punjab, India; 3 Division of Pulmonary and Critical Care Medicine, University of Michigan Health System, Ann Arbor, Michigan, United States of America; University of Georgia, UNITED STATES

## Abstract

It is increasingly evident that cytokines and growth factors produced in the decidua play a pivotal role in the regulation of the local immune microenvironment and the establishment of pregnancy. One of the major growth factors produced in the decidua is vascular endothelial growth factor (VEGF), which acts not only on endothelial cells, but also on multiple other cell types, including macrophages. We sought to determine whether decidua-derived VEGF affects macrophage recruitment and polarization using human endometrial/decidual tissue samples, primary human endometrial stromal cells (ESCs), and the human monocyte cell line THP1. *In situ* hybridization was used for assessment of local VEGF expression and immunohistochemistry was used for identification and localization of CD68-positive endometrial macrophages. Macrophage migration in culture was assessed using a transwell migration assay, and the various M1/M2 phenotypic markers and VEGF expression were assessed using quantitative real-time PCR (qRT-PCR). We found dramatic increases in both VEGF levels and macrophage numbers in the decidua during early pregnancy compared to the secretory phase endometrium (non-pregnant), with a significant increase in M2 macrophage markers, suggesting that M2 is the predominant macrophage phenotype in the decidua. However, decidual samples from preeclamptic pregnancies showed a significant shift in macrophage phenotype markers, with upregulation of M1 and downregulation of M2 markers. In THP1 cultures, VEGF treatment significantly enhanced macrophage migration and induced M1 macrophages to shift to an M2 phenotype. Moreover, treatment with conditioned media from decidualized ESCs induced changes in macrophage migration and polarization similar to that of VEGF treatment. These effects were abrogated by the addition of a potent VEGF inhibitor. Together these results suggest that decidual VEGF plays a significant role in macrophage recruitment and M2 polarization, and that inhibition of VEGF signaling may contribute to the shift in macrophage polarity observed in different pregnancy disorders, including preeclampsia.

## Introduction

During embryo implantation the uterine endometrium becomes decidualized, an inflammatory process that involves not only transformation of endometrial stromal cells into specialized secretory cells, but also an influx of a multitude of immune cells [1]. These immune cells are believed to play a central role in setting the balance between immune tolerance and proinflammatory responses, which is critical for proper implantation and establishment of a viable pregnancy [2]. Excess inflammation, resulting from a failure to maintain this balance, is linked to pregnancy loss and disorders of pregnancy, such as preeclampsia [3–5].

While multiple lineages of immune cells are prevalent at the site of implantation, macrophages are one of the most abundant, accounting for 10–15% of cells in the uterine decidua throughout pregnancy [6]. Although contradictory reports exist [7, 8], most studies support the proposition that macrophages are polarized toward 2 different phenotypes: M1 and M2. M1 macrophages produce pro-inflammatory cytokines, present antigens, and produce nitric oxide and reactive oxygen species, whereas M2 macrophages are responsible for immune tolerance and tissue remodeling [9]. Both M1 and M2 macrophages are present in the uterine decidua during pregnancy but their relative numbers vary; after an initial inflammatory phase, when M1 macrophages predominate, decidual macrophages have a predominantly M2 phenotype until the onset of parturition [10]. Emerging evidence suggests that macrophage homing and phenotype switching is of paramount importance for successful pregnancy and that dysregulation of macrophage polarity is linked to several disorders of pregnancy, including recurrent pregnancy loss and preeclampsia [10–14]. However, little is known about the factors that regulate macrophage differentiation and polarization during pregnancy.

VEGF is named for its function in angiogenesis, but it also has many non-endothelial cell functions [15]. VEGF is produced by macrophages [16], decidualized endometrial cells [17], and trophoblasts [18] and is critical for the process of implantation [19]. VEGF acts primarily through two different transmembrane receptors, Flt1 (VEGFR1) and KDR (VEGFR2), but also binds to a soluble form of the Flt1 receptor (sFlt1) that is created by alternative splicing of Flt1 mRNA [20]. The sFlt1 receptor acts as a VEGF inhibitor, as it binds to VEGF with high affinity but has no capability to mediate intracellular signaling due to its lack of a transmembrane component [21]. Excess production of placental sFlt1, leading to impaired VEGF signaling, is known to be associated with several pregnancy complications, including preeclampsia [22, 23]. VEGF has been implicated in the regulation of macrophage functions, including migration and polarity, in other tissues [24–27]. However, although decidual macrophages are known to have unique characteristics [28, 29], the roles of VEGF signaling in the regulation of macrophage functions during pregnancy are poorly understood.

Thus, the purpose of this study was to investigate the roles of VEGF in macrophage recruitment and polarization in early pregnancy. Using ex-vivo human tissues, we established a strong correlation between VEGF levels and macrophage number. Furthermore, using well-established in vitro model systems, we demonstrate that both endogenous decidual VEGF and exogenous recombinant VEGF can stimulate macrophage migration and polarization to the M2 phenotype. Additionally, we show that the balance of M1/M2 macrophages is altered in preeclampsia. These results suggest that VEGF likely plays a role in decidual macrophage recruitment and polarization and that changes in macrophage polarity are associated with preeclampsia.

## Materials and methods

### Human subjects

Tissue samples were collected after obtaining informed consent under protocols approved by the Wayne State University and Stanford University IRBs on the use of Human Subjects in Medical Research for our previous studies [22, 30, 31]. Briefly, secretory phase endometrium was obtained from subjects undergoing hysterectomy for benign indications, such as fibroids or chronic pelvic pain, in the age group between 28 and 39 years during the secretory phase of the cycle, as described previously [31]. Determination of the stage of the menstrual cycle was based on the day of last menstruation and histological evaluation by pathologists [31]. Full-thickness endometrial OCT blocks (2–3 mm) and cryosections (10μm) were prepared for in situ hybridization studies and RNA extracted for qPCR analysis as described below (n = 5). Decidual samples from early pregnancy (5–9 weeks) were collected from subjects undergoing elective termination of pregnancy following the same procedures as described previously [30, 32]. After dilation and curettage, deciduas were separated from chorionic villi and other fetal tissues under a dissecting microscope and processed for RNA extraction and preparation of OCT blocks. Decidual samples from term pregnancies were used from a cohort of 19 preeclamptic and 24 gestational age-matched controls, and 4 samples per group were used for this study. Decidual tissues were carefully separated from attached placentas or placental bed biopsies and used for RNA extraction as described below [33]. Details of clinical evaluations have been reported previously [33]. Preeclamptic women had systolic blood pressures of ≥ 140 mm Hg and diastolic blood pressures of ≥ 90 mm Hg more than once, with proteinuria of ≥ 300 mg/24h [33]. All endometrial/decidual samples were stored at -80 °C and processed for RNA extraction, *in situ* hybridization, and immunohistochemistry as described below.

### *In situ* hybridization

In situ hybridization (ISH) for detection of human VEGF mRNA expression was performed in frozen endometrial/decidual sections following the same procedures and probes as described previously [22, 31, 34]. ^35^S-UTP-labeled (PerkinElmer, Hopkinton, MA) sense and antisense riboprobes from VEGF cDNA templates were prepared with the MAXIscript in vitro transcription kit from Applied Biosystems (Waltham, MA), and purified using mini Quick Spin TM RNA Columns (Roche, Indianapolis, IN). Sections were fixed in 4% paraformaldehyde and hybridized with 5x10^6^ cpm/ml S^35^-labeled sense and antisense probes. Sections treated with RNase-A or treated with the sense probe were used as negative controls. Hybrids resistant to RNase-A were detected by autoradiography, and sections were post-stained with hematoxylin. Images were taken on a Zeiss Axioskop 2 microscope equipped with a Zeiss AxioCam camera (Carl Zeiss).

### Immunohistochemistry

Immunohistochemistry (IHC) was performed using 10 μm OCT-embedded frozen tissue sections fixed with 4% paraformaldehyde following our previously published methods [22, 35]. After washing and incubation with horse blocking serum, the sections were incubated with a mouse monoclonal primary antibody to CD68 (1:200, Agilent, Santa Clara, CA) overnight at 4°C, followed by blocking and incubation with a biotinylated anti-mouse secondary antibody (Vector Laboratories, Burlingame, CA) at room temperature. The sections were visualized by immunofluorescence imaging using fluorescein-labeled avidin and Vectashield mounting medium with DAPI (Vector Laboratories).

### RNA extraction and quantitative real time PCR (qPCR)

Total RNA extraction and qPCR were performed following the same methods as previously published [35]. Total RNA from tissues/cells was isolated using TRIZOL (Invitrogen, Carlsbad, CA), treated with DNase (Roche), and purified using RNeasy Spin Columns (Roche). Two micrograms of RNA were used for cDNA synthesis using a kit from Promega (Madison, WI). Real-time PCR was performed with the 7500 Fast instrument (Applied Biosystems) using SYBR Green PCR master mix (Applied Biosystems) and the respective primer pairs for each gene (sequences presented in Table 1). All experiments were performed in triplicate, and data were normalized against beta-actin. Water was used as a negative control, and the size and sequence of each PCR product were used for validation of the results. The relative expression of mRNAs for various genes is presented as fold change using the 2^^ (-ΔΔCT)^ method as previously described [36].

**Table 1:** Primers for quantitative real-time PCR.

Gene		Primer
**M1**	**CXCL10**	F 5’-GAAAGCAGTTAGCAAGGAAAGGTC-3’ R 5’-ATGTAGGGAAGTGATGGGAGAGG-3’
	**CCR7**	F 5’-TGGTGGTGGCTCTCCTTGTC-3’ R 5’-TGTGGTGTTGTCTCCGATGTAATC-3’
	**IL-12**	F 5’-AAAGGACATCTGCGAGGAAAGTTC-3’ R 5’-CGAGGTGAGGTGCGTTTATGC-3’
**M2**	**CD206**	F 5’-ACCTCACAAGTATCCACACCATC-3’ R 5’-CTTTCATCACCACACAATCCTC-3’
	**CD163**	F 5’-GTCGCTCATCCCGTCAGTCATC-3’ R 5’-GCCGCTGTCTCTGTCTTCGC-3’
	**CCL-17**	F 5’-GAGCCATTCCCCTTAGAAAG-3’ R 5’-AGGCTTCAAGACCTCTCAAG-3’
**VEGFR1**		F 5’ -TGGCAGCGAGAAACATTCTTTTAT-3’ R 5’-CAGCAATACTCCGTAAGACCACAC-3’
**VEGFR2**		F 5’-CTCTTGGCCGTGGTGCCTTTG-3’ R 5’-GTGTGTTGCTCCTTCTTTCAAC-3’
β-**Actin**		F 5’-ATTGCCGACAGGATGCAGAA-3’ R 5’-GCTGATCCACATCTGCTGGAA-3’

### THP-1 cell culture

Human monocytic THP-1 cells (ATCC, Rockville, MD) were cultured in RPMI-1640 medium (Life Technologies, Carlsbad, CA) supplemented with 10% FBS, 2mM L-Glutamine and 1% antibiotics (penicillin, streptomycin and amphotericin-B) (Sigma-Aldrich, St. Louis, MO). Differentiation of THP-1 cells into M0, M1, and M2 macrophages was performed as previously described [37]. Briefly, THP-1 cells were differentiated into M0 macrophages by incubation with 100 nM phorbol 12-myristate 13-acetate (PMA) for 48 h (Sigma-Aldrich). Once the cells were adherent, they were transferred to PMA-free media to obtain resting macrophages (M0). These cells were then polarized to M1 macrophages by incubation with LPS (100 ng/ml) and IFN-γ (20 ng/ml) for 48 h and M2 macrophages by incubation with IL-4 (100ng/ml) for 48 h. In a subset of experiments, to determine the effective dose of VEGF, M1 cells were treated with different doses of rhVEGF (0.1–20 μg/mL) (R&D systems, Minneapolis, MN). Based on the results of this experiment, the cells were treated with/without 4 μg/mL of rhVEGF (most effective dose) for subsequent experiments.

### *In vitro* decidualization of endometrial stromal cells

Primary endometrial stromal cells were isolated from endometrial tissues obtained from normally cycling women undergoing hysterectomy for benign conditions as indicated above and described previously [38–40]. Briefly, endometrial tissues were digested with collagenase, and stromal cells were separated from the epithelium and cultured in DMEM (Gibco, Gaithersburg, MD) supplemented with 5 μg/ml insulin (Sigma, St. Louis, MO) and 10% heat-inactivated charcoal-stripped fetal bovine serum (FBS, Gibco). At passages 3–4, the cells were grown to confluence and then decidualized with 10 nm estradiol, 1 μm progesterone, and 20 ng/ml epidermal growth factor in serum-free and insulin-free decidualization medium (75% DMEM, 25% MCDB-105, 50 μg/ml ascorbic acid, 1 mg/ml BSA, 5 μg/ml transferrin) (Sigma) [40]. Decidualization occurred after 7–14 days in culture and was confirmed by documenting increased secretion of insulin-like growth factor-binding protein-1 into the medium, as described previously [38, 39]. Cells treated with decidualization medium only (without steroid hormones) were used as controls. RNA and conditioned media from both decidualized and non-decidualized cells were collected. The conditioned media were concentrated ten-fold using a Centricon filter (10 kD MWCO, Millipore, Billerica, MA) and used in subsequent experiments with or without the VEGF inhibitor sFlt1 (1.5 μg/ml, R&D systems) to examine the role of VEGF secreted into the conditioned media on differentiation and migration of macrophages.

### *In vitro* cell migration assay

*In vitro* cell migration assays were performed in 24-well plates using transwell inserts with 8 μm pores (Corning, Corning, NY) following the procedures described previously [41]. Briefly, M0 cells were seeded (5 X 10^4^ cells/insert) onto the upper well of the chamber, and the inserts were placed in 24-well cell culture plates (Corning) containing 500 μL of RPMI-1640 medium with or without 2 μg/ml rhVEGF, and incubated at 37°C in 5% CO2 for 16 hours. In another set of experiments, the cells were incubated in RPMI-1640 medium with concentrated supernatants from decidualized endometrial stromal cells and with or without sFlt1 (1.5 μg/ml; R&D systems), as described above. Concentrated supernatants from nondecidualized stromal cells were used as controls. The cells in the upper chambers were removed carefully with a wet cotton swab. The migrated cells on the bottom surface were stained with Diff-Quick Hema3 stain following the manufacturer’s instructions (Fisher Scientific, Kalamazoo, MI), and six evenly spaced fields of cells were counted in each well using a Leica DMIRES2 microscope.

### Statistical analysis

Comparisons were made using an analysis of variance (ANOVA) with Tukey post-test or Students’ t-test, as appropriate. A p-value of equal to or less than 0.05 was considered statistically significant. Statistical analyses were performed using GraphPad Prism (GraphPad Software, Inc.; La Jolla, CA, USA) or Microsoft Excel (Microsoft Corporation; Redmond, WA, USA), as described previously.

## Results

### Alteration in decidual macrophage polarity in normal and preeclamptic pregnancies

**Fig 1:**
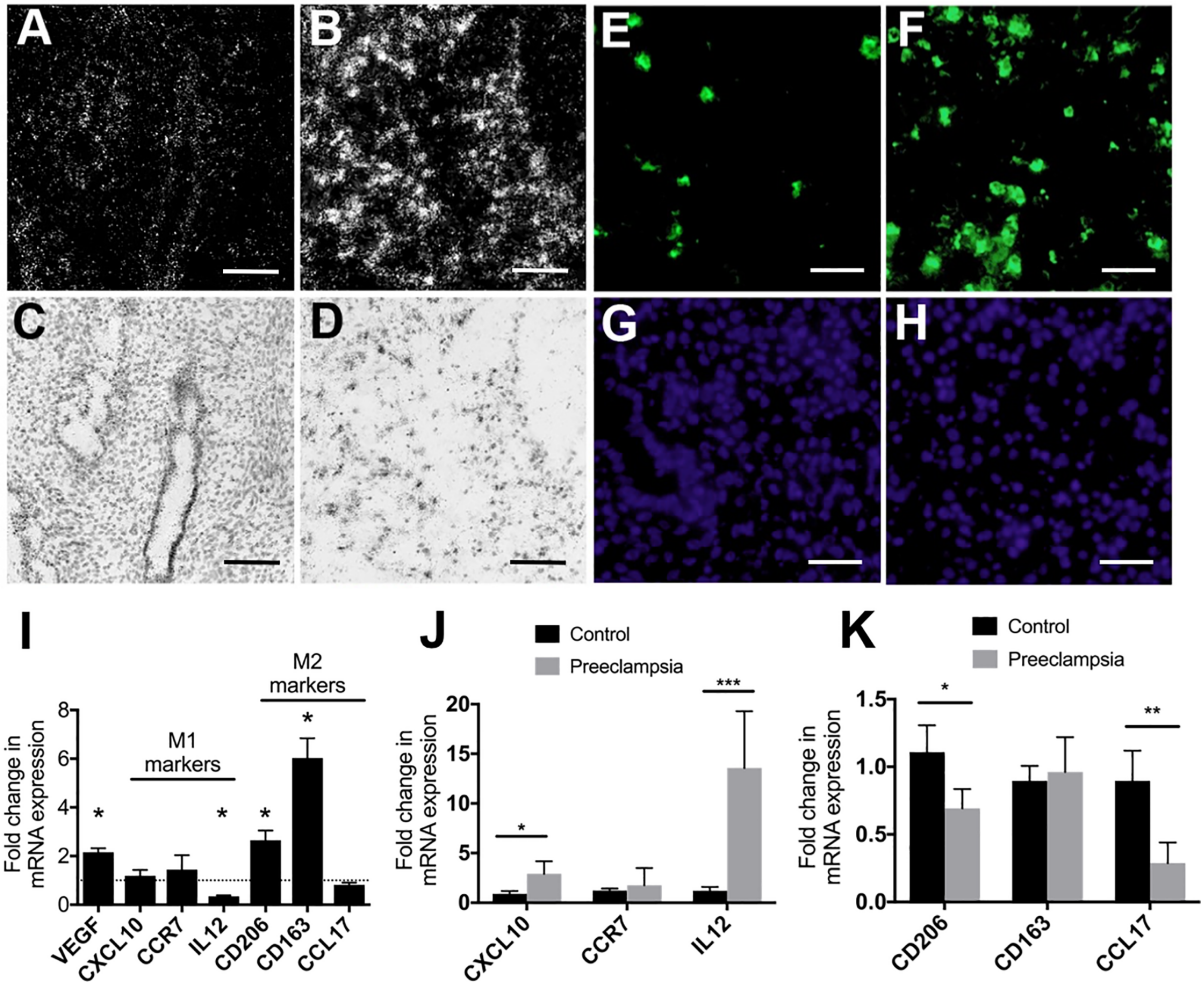
Increase in decidual VEGF expression is associated with increase in macrophage number and dramatic upregulation of M2 markers in the decidua. A-D, representative photomicrographs (in situ hybridization; A-B, dark-field images; C-D, bright-field images) show a marked increase in VEGF mRNA expression in first trimester decidua (B, D) as compared with secretory phase endometrium (A, C). Immunofluorescence (E-F, CD68 immunostaining; G-H, DAPI staining) show a concomitant increase in CD68-positive macrophages in first trimester decidua (F) as compared to secretory phase endometrium (E). Scale bars: A-D, 100 μm and E-H, 40 μm. I, qPCR analysis of VEGF and M1 (CXCL10, CCR7, and IL12) and M2 (CD206, CD163, and CCL17) markers in first trimester decidual tissue compared with secretory phase endometrium. J-K, qPCR analysis of M1 (J) and M2 (K) markers in decidual samples from preeclamptic pregnancies compared with gestational-age matched controls. Note the significant increase in M1 markers and decrease in M2 markers in decidual samples from preeclamptic pregnancies. Graphs represent means ± standard errors; * = p ≤0.05, ** = p <0.01, and *** = p <0.001.

To examine the potential importance of VEGF in macrophage function in the decidua, we first determined VEGF levels and macrophage numbers in decidua and secretory phase endometrium. Using *in situ* hybridization we found that VEGF mRNA levels were dramatically higher in the decidua than in the secretory phase endometrium (Fig 1A–1D), while immunostaining revealed a concomitantly greater number of CD68-immunopositive macrophages in the decidual samples (Fig 1E–1H). Measurement of total RNA by real-time PCR also revealed significantly higher VEGF levels in decidual tissue (Fig 1I), and analysis of macrophage phenotypic markers indicated significantly lower levels of the M1 marker IL-12 and significantly higher levels of the M2 markers CD206 and CD163, suggesting that M2 is the predominant phenotype of decidual macrophages in early pregnancy. However, qPCR analysis of RNA samples from deciduas of preeclamptic and gestational age-matched control samples revealed significantly greater levels of M1 markers (CXCL10 and IL12) and significantly lower levels of M2 markers (CD206 and CCL17) in the decidual samples from preeclamptic pregnancies (Fig 1J and 1K), suggesting a shift in decidual macrophage polarity (from M2 to M1) in preeclamptic pregnancies.

### Differentiation of THP-1 monocytes into polarized macrophages

**Fig 2:**
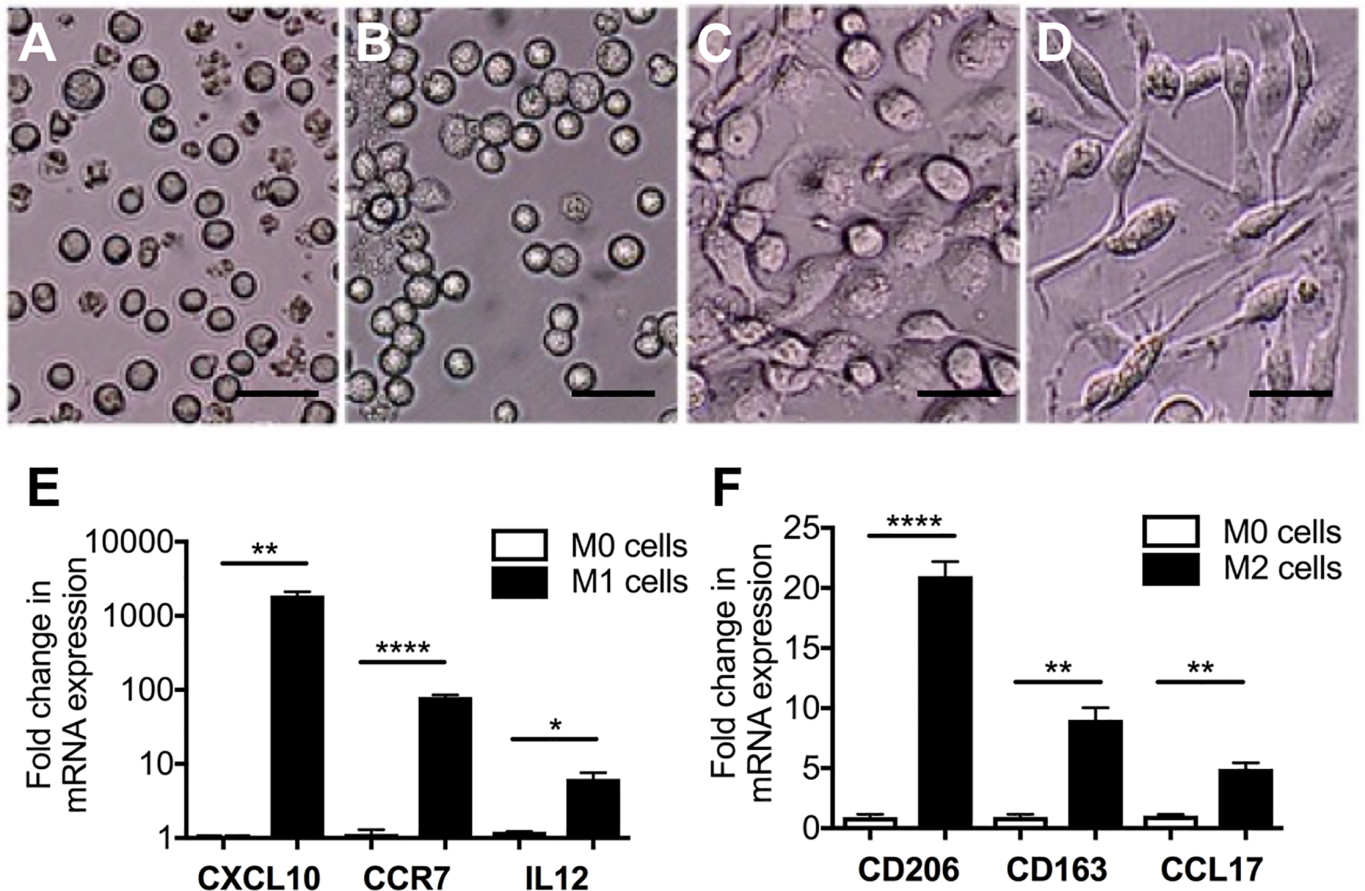
THP1 cells differentiated into polarized macrophages display characteristic morphology and markers of polarization. A-D, photomicrographs showing characteristic morphologies of THP1 cells (A), and differentiated M0 (B), M1 (C), and M2 (D) macrophages. M1 macrophages (C) appear flattened as compared with M0 cells (B), and M2 macrophages take on a more elongated shape (D). E-F, qPCR quantification of M1 (E) and M2 (F) macrophage markers in THP1-derived M1 and M2 cells, respectively, relative to M0 cells. The expression of M1 and M2 mRNA markers is consistent with the observed morphologies of the M1 (C) and M2 (D) macrophages. Graphs represent means ± standard error. Scale bar = 30 μm; * = p < 0.05, ** = p <0.01, and **** = p <0.0001.

First we standardized and characterized the ability of THP-1 monocyte cells to undergo differentiation into different macrophage phenotypes with known stimuli to further study the effect of VEGF on macrophages in a culture model. THP-1 cells exposed to PMA for 48 hours became adherent (Fig 2B), a characteristic of M0 cells. These cells acquired a rounded and flat M1 phenotype (Fig 2C) after exposure to LPS and IFN- γ. The M0 cells became highly elongated following treatment with IL-4 (Fig 2D), a characteristic of the M2 phenotype [42]. Quantitative real-time PCR was then used to measure the mRNA markers of the M1 (CXCL10, CCR7, and IL12) and M2 (CD206, CD163, and CCL17) phenotypes. Consistent with the observed morphological changes, there were significantly greater levels of CXCL10 (1,600-fold), CCR7 (78-fold), and IL12 (13-fold) in M1 cells (Fig 2E) and significantly greater levels of CD206 (24-fold), CD163 (8-fold), and CCL17 (5-fold) in M2 cells (Fig 2F) relative to M0 cells.

### VEGF treatment suppresses M1 markers and induces M2 markers in polarized macrophages

**Fig 3:**
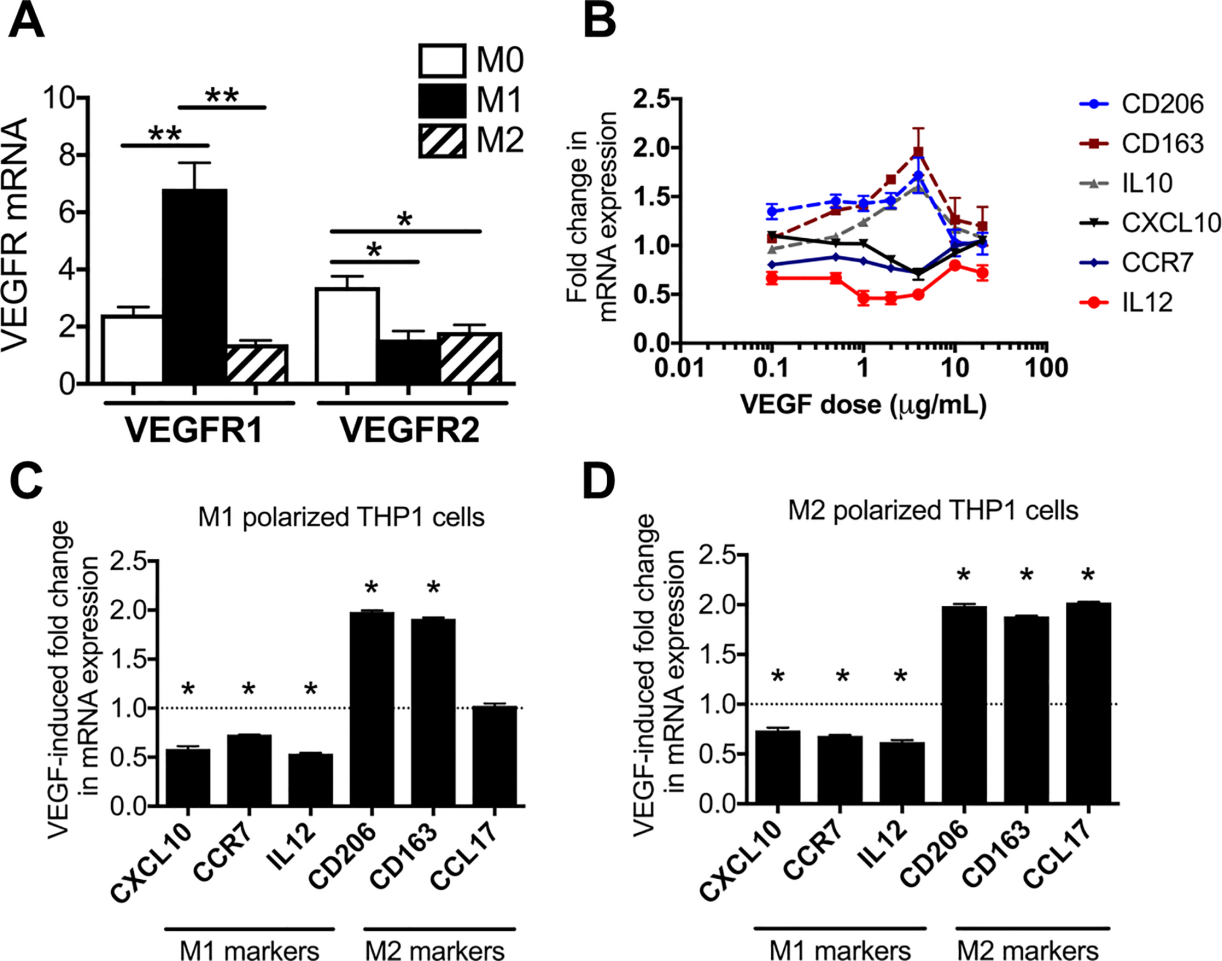
VEGF treatment suppresses M1 markers and induces M2 markers in polarized macrophages. A, qPCR analysis of M0, M1, and M2 cells shows that all three express both VEGF receptors 1 (VEGFR1) and 2 (VEGFR2). Note the highly significant increase in expression of VEGFR1 in M1 cells. B, initial dose-response study showing the effect of increasing concentrations of rhVEGF (0.1–20 μg/mL). VEGF induces an upregulation of M2 markers and downregulation of M1 markers in THP1-derived M1 cells, with highest response seen at 4 μg/mL. C-D, VEGF treatment at 4 μg/mL caused an increase in M2 markers and a decrease in M1 markers in both THP1-derived M1 (C) and M2 (D) macrophages. Graphs represent means ± standard errors; * = p < 0.05, ** = p <0.01.

To evaluate VEGF functional capabilities in macrophages, we first examined expression of the two major VEGF receptors, VEGFR1 and VEGFR2, in differentiated, polarized THP-1 cells. qPCR analysis revealed expression of both VEGFR1 and VEGFR2 in all three phenotypes, but expression of VEGFR1 was significantly higher in M1 cells than in either M0 or M2 cells (Fig 3A). It may be noted that VEGFR2 was downregulated in both M1 and M2 cells relative to M0 cells (Fig 3A). Interestingly, in an initial dose-response study, treatment of M1 cells with VEGF caused a dramatic upregulation of M2 markers and downregulation of M1 markers, with the maximum effect occurring at 4 μg/mL of VEGF (Fig 3B). Consistent with these results, treatment of M1 and M2 cells with 4 μg/mL of VEGF caused significant upregulation of most M2 markers and significant downregulation of M1 markers in both M1 and M2 cells (Fig 3C and 3D), suggesting that VEGF may play a role in the shift from the M1 to the M2 macrophage phenotype.

### Decidua-derived VEGF may play a role in polarization of macrophages to the M2 phenotype

**Fig 4:**
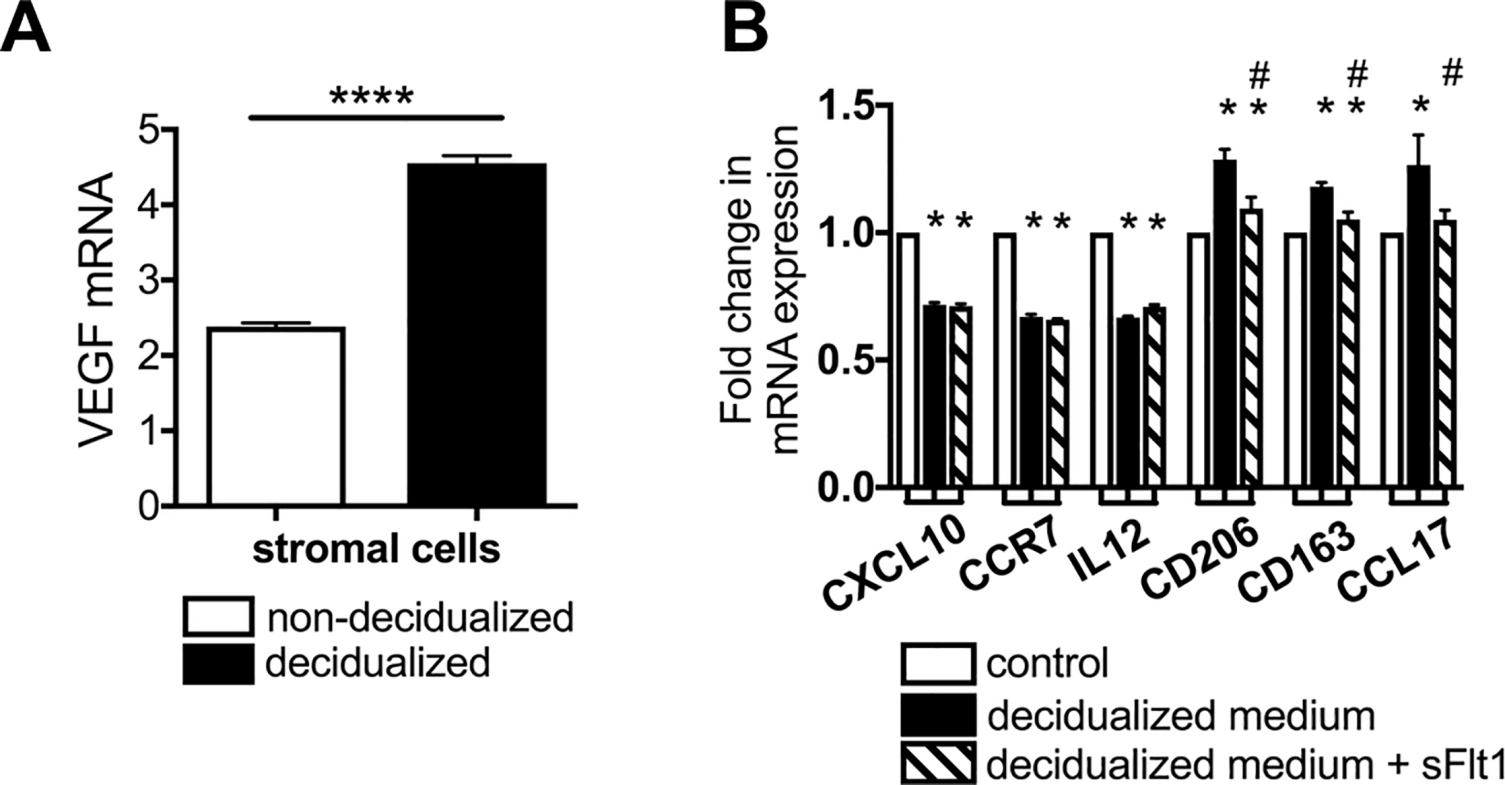
Decidual endometrial stromal cells produce VEGF and stimulate M2 macrophage polarization. A, qPCR analysis of VEGF mRNA expression in decidualized and non-decidualized stromal cells. VEGF expression is highly significantly increased following decidualization. B, expression of M1 and M2 mRNA markers following treatment with conditioned medium from decidualized/non-decidualized stromal cells, with or without the VEGF inhibitor sFlt1. Decidualized stromal cell conditioned medium induces a significant decrease in all M1 markers and a significant increase in all M2 markers, but the effect of the conditioned medium on M2 markers was abrogated by sFlt1 treatment, suggesting a potential role for VEGF in M2 polarization. Graphs represent means ±standard errors; * = p < 0.05 compared to controls; # = p<0.05 compared to decidualization medium; **** = p <0.0001.

Next, to determine if VEGF produced in decidualized stromal cells can influence macrophage function, we treated THP-1-differentiated M1 cells with conditioned medium from decidualized human endometrial stromal cells (HESCs). Decidualized primary cultures of HESCs produced nearly double the amount of VEGF mRNA (highly significant) as compared to non-decidualized HESCs (Fig 4A). Notably, treatment of M1 cells with decidualized HESC-conditioned medium resulted in a significant decrease in all M1 markers (CXCL10, CCR7 and IL12), and a significant increase in all M2 markers (CD206, CD163 and CCL17) (Fig 4B), similar to the effect of VEGF treatment on M1 cells (Fig 3C). The effect of decidualized HESC conditioned medium on M2 markers was abrogated by the addition of a potent VEGF inhibitor, sFlt1, to the medium (Fig 4B), suggesting a role for decidua-derived VEGF in the shift from M1 to M2 macrophage phenotype. However, the addition of sFlt1 did not alter the effect of decidualized HESC-conditioned medium on M1 markers (Fig 4B), suggesting that factors other than VEGF produced by decidualized stromal cells may be involved in the suppression of the inflammatory cytokines and chemokines produced by M1 cells.

### Decidua-derived VEGF may facilitate monocyte/macrophage recruitment/migration into the decidua

**Fig 5:**
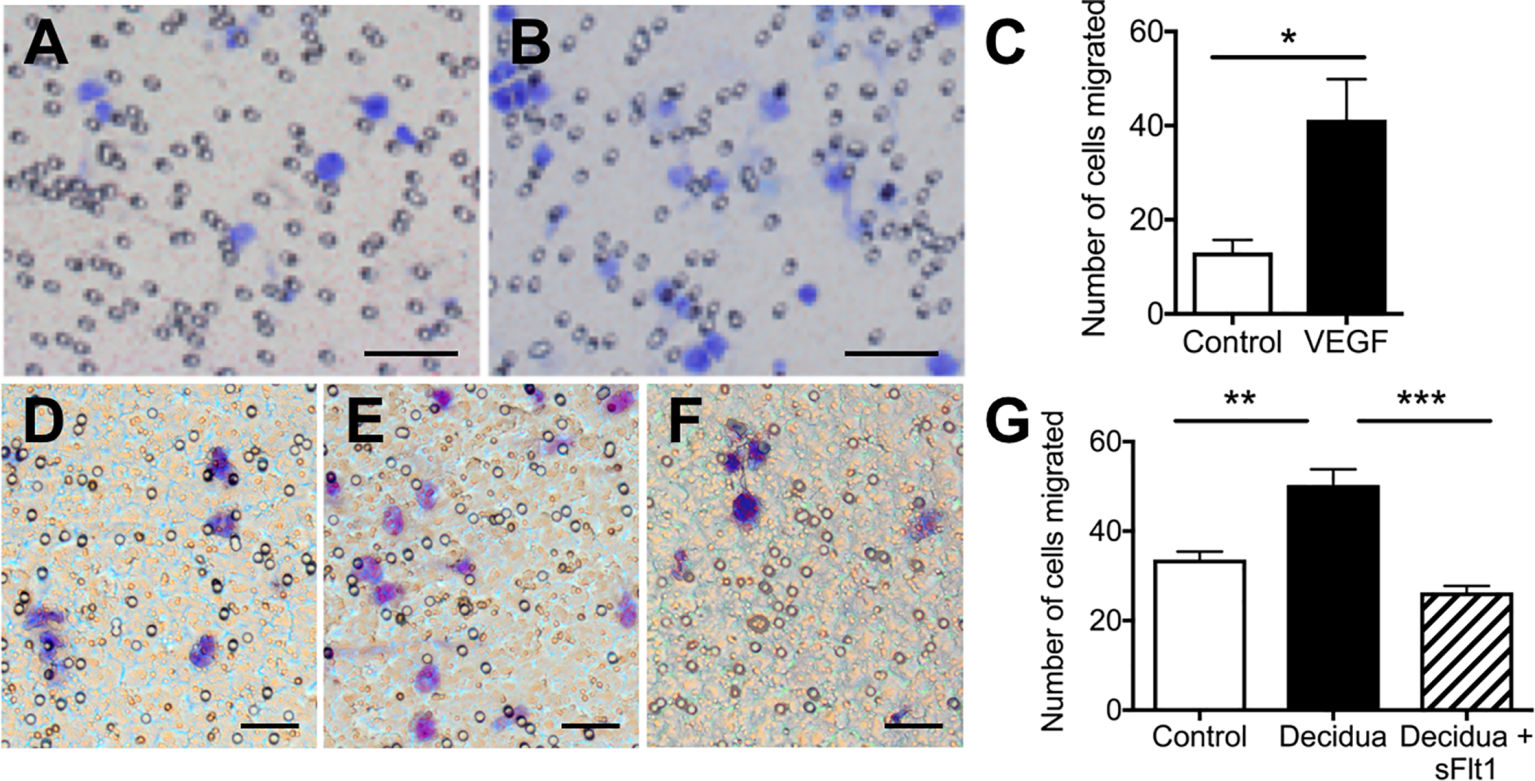
VEGF produced by decidualized endometrial stromal cells stimulate macrophage migration. A-C, transwell migration assay showing migration of M0 cells without (A) or with (B) VEGF treatment (2 μg/mL). Bar graph (C) shows quantitative estimation of the number of migrated cells. Addition of VEGF to the medium in the lower chambers induced about a 3-fold increase in migration of cells (B and C). D-G, transwell migration assay of M0 cells; D, treatment with non-decidualized stromal cell-conditioned medium; E-F, treatment with decidualized stromal cell-conditioned medium in the absence (E) or presence (F) of the VEGF inhibitor sFlt1; G, quantification of migrated cells. Note that decidualized stromal cell-conditioned medium significantly stimulated migration of M0 cells (E and G) but the effect was completely abrogated by the addition of sFlt1 (F), suggesting that VEGF secreted into the conditioned medium may play a role in stimulating the migration of these cells. Graphs represent means ± standard errors; * = p<0.05, ** = p<0.01, *** = p<0.001. Scale bars represent 50 μm.

The effect of VEGF on migration of undifferentiated macrophages (THP-1 M0 cells) was examined using the transwell migration assay system. VEGF treatment resulted in a significant increase (about three-fold) in the migration of M0 cells toward the VEGF-containing medium (Fig 5A–5C). Furthermore, treatment of M0 cells with conditioned medium from decidualized primary cultures of HESCs also significantly stimulated macrophage migration (Fig 5D–5G), and this effect was abrogated by the addition of sFlt1 (a potent VEGF inhibitor) to the conditioned medium (Fig 5F and 5G), suggesting that VEGF may play a role in migration of monocytes/macrophages towards VEGF-producing decidual stromal cells.

## Discussion

Emerging evidence suggests that macrophage homing to the decidua and phenotype switching is of paramount importance in the establishment and maintenance of pregnancy [43–45]. Because of their high degree of plasticity, macrophage phenotype switching is greatly influenced by the changing decidual microenvironment at different stages of normal pregnancy and in disease states. Decidual macrophages have been reported to be predominately of the M2 phenotype [29, 46, 47], consistent with the immune tolerant environment at the maternal-fetal interface. A shift in the balance between the two phenotypic classes toward the M1 phenotype has been reported in several pregnancy disorders, including preeclampsia [11–13, 48]. Although several factors have been found to regulate M1 and M2 polarization during pregnancy [49, 50], the molecular mechanisms of macrophage recruitment and polarization in the decidua are not fully understood. In this study, we show that VEGF levels and macrophage numbers positively correlate in the decidua in early pregnancy. Consistent with previous findings, we show that most decidual macrophages in early pregnancy have an M2 phenotype. We have also demonstrated that VEGF can polarize THP-1-derived macrophages toward the M2 phenotype and that VEGF produced by decidualized endometrial stromal cells mimics this effect, and the data are consistent with VEGF or VEGF-dependent factors acting as chemoattractants for macrophages. We further demonstrate an increase in M1 markers in the deciduas of patients with preeclampsia. Taken together, these results indicate that decidual VEGF plays an important role in recruitment/migration and polarization of macrophages in early pregnancy and a potential role in the dysregulation of macrophage polarity in the pathogenesis of preeclampsia.

THP-1 cells are a human monocytic cell line first characterized in 1980 that have since been extensively utilized for studying monocyte and macrophage differentiation and function [51]. THP-1 cells can be polarized into M1 and M2 macrophages using the same stimuli that are used for primary human monocyte/macrophage cells in culture [52]. M1 and M2 macrophages exist on a spectrum, and it remains unclear which markers are most specific for identifying M1 and M2 polarization under different experimental conditions [7, 53]. In this study, we evaluated several M1 and M2 markers that are among the most well-studied [7], including markers that have been specifically identified in decidual macrophages, such as CD206 [28, 29, 44]. Consistent with our findings, a recent study also showed that CD163 increases when primary human decidual macrophages are exposed to the M2 stimuli [54], and both CD163 and CD206 are expressed in decidual macrophages in vivo [7, 29, 54]. Based on these findings along with published experimental guidelines for macrophage investigation, we selected CD206 and CD163, as well as CCL17, a chemokine produced primarily by M2 macrophages, as markers of M2 polarity [7, 9]. The M1 markers examined were the chemokine CXCL10, the cytokine IL-12, and the chemokine receptor CCR7, which are widely used to demonstrate classical activation of macrophages [7, 55]. Consistent with prior reports, we have demonstrated that THP-1 cells/macrophages express both VEGFR1/Flt1 and VEGFR2, [56–58]. Our results are also consistent with earlier reports showing that VEGFR1 is highly expressed in M1 but only at low levels in M2 macrophages and that there is significant upregulation of VEGFR1 expression during differentiation into the M1 phenotype [59, 60], suggesting that VEGF can directly influence macrophage function, likely mediated by the VEGFR1/VEGF axis.

Isolated human endometrial stromal cells undergo decidualization in culture in response to ovarian steroid hormones with similar morphological and biochemical endpoints as those for decidualization in vivo, and this model has been extensively used in studies of decidual function [38, 39]. Similarly, several previous studies have demonstrated VEGF-dependent chemotactic responses by monocytes/macrophages, mediated through Flt1 [61–63]. Our results show that conditioned medium from decidualized endometrial stromal cells contains VEGF and promotes both macrophage chemotaxis and polarization to the M2 phenotype, suggesting that endogenous VEGF levels produced by decidualized endometrial stromal cells are enough to stimulate macrophage recruitment/migration and polarization. Furthermore, these effects were significantly, but not fully, mitigated by the addition of the VEGF inhibitor sFlt1, indicating that VEGF in the conditioned medium is one of the major contributing factors but that other factors may also be involved. Although there is some suggestion in the literature that VEGF can polarize tumor-associated macrophages to the pro-tumor M2 phenotype [64], our results for the first time suggest a potential role for decidual VEGF in macrophage recruitment/migration and M2 polarization. However, in a recent study in a skin cancer model, Linde et al. found that VEGF alone was not sufficient to promote M2 polarization in vitro, but required co-stimulation with IL-4 and IL-10 [26]. The difference may be explained by the fact that Linde et al. used bone marrow-derived macrophages and a lower concentration of recombinant VEGF for their studies. Indeed, we found that increasing doses of VEGF induced a more robust shift in polarity from the M1 to M2 phenotype up to a maximum concentration of 4 μg/mL, at which point there is a paradoxical shift back toward the M1 phenotype. Although the reasons for this reversal are unclear, our previous studies and other reports suggest that VEGF can exhibit highly concentration-dependent effects when examined in the tissue environment and can act pleiotropically, potentially performing different functions at different threshold concentrations [22, 65].

Decidual macrophages are believed to exist on a continuum from M1 to M2, with predominance of the M2 phenotype throughout the majority of pregnancy [10, 66]. Several factors have been shown to regulate M1 versus M2 polarization during pregnancy and many of these factors, including the most potent M2 polarization factor, M-CSF, are known to stimulate VEGF production in macrophages and other cells [67]. This suggests that VEGF may serve as a common mediator in their signaling pathways and that the effects depend on the local level of VEGF production, as described above.

Additionally, VEGF is known to act as a chemoattractant for monocytes and macrophages and plays a major role in the recruitment of macrophages into hypoxic and inflammatory tissues [68]. Although, the precise mechanism by which VEGF induces migration of macrophages is not clearly understood, VEGF has been shown to stimulate cellular migration in other cell types by activating multiple signaling molecules downstream of Flk1/KDR and Flt1 [69–72]. Consistent with previous studies [61–63], our results show that VEGF-induced migration of macrophages is mediated at least in part through increased expression of Flt1. Together, our results suggest that VEGF produced by decidualized endometrial stromal cells may stimulate decidual macrophage recruitment into the hypoxic decidua at the maternal-fetal interface. Because knockdown of VEGFR1 has been shown to block macrophage recruitment into tumor tissues [73], and as our study shows that VEGFR1 is highly expressed on M1 but not on M2 macrophages, we speculate that high levels of VEGF produced by the decidual cells at the maternal-fetal interface may stimulate migration of M1 macrophages to this area and promote their differentiation into M2 macrophages.

We and others have previously reported that VEGF levels increase in the deciduas of patients with preeclampsia [22, 74]. Despite this increase in VEGF, which should act to induce M2 polarity, we found that M1 macrophage markers predominate in the preeclamptic decidua. This is consistent with the observation by Medeiros *et al*., who showed increased numbers of M1 macrophages in patients with preeclampsia [12], and other studies showing increased numbers of total macrophages [13] and decreased numbers of M2 macrophages in preeclampsia [14]. This apparent contradiction may be explained by the results of our previous study indicating that excess decidual VEGF production induces sFlt1 production in the invading trophoblast cells at the decidual-placental interface, ultimately resulting in insufficient VEGF signaling at the decidual-placental interface and contributing to the shift toward the M1 phenotype.

**Fig 6:**
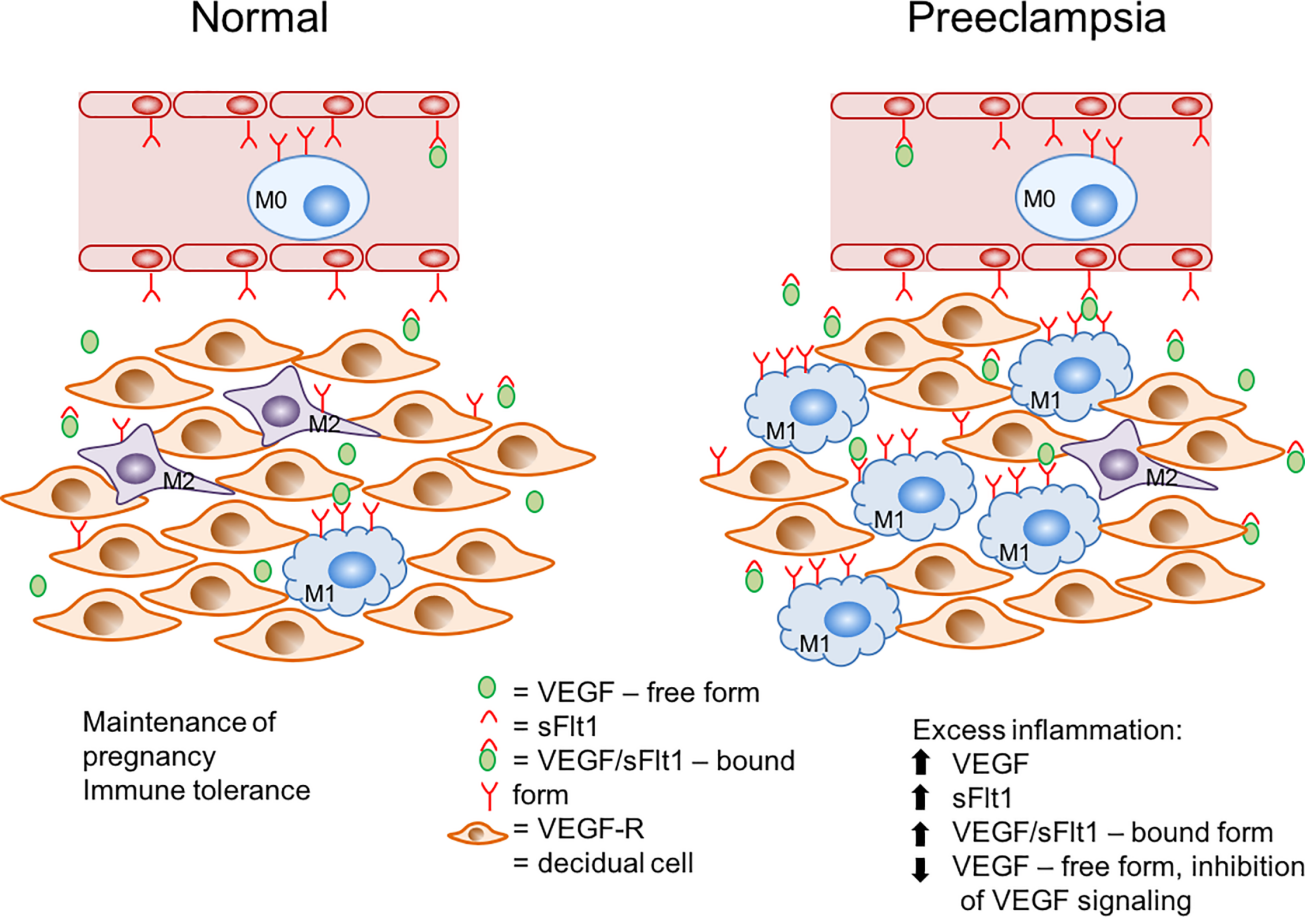
Model of VEGF action on decidual macrophages. Decidual stromal cells produce VEGF, which acts to recruit monocytes and tissue macrophages to the site of implantation in normal pregnancy. It also acts to polarize macrophages to the M2 phenotype. In preeclampsia, however, there is an increase in VEGF, which may enhance recruitment of macrophages to the decidua. Excess VEGF at the implantation site in preeclamptic pregnancies may also stimulate excess production of sFlt1 by trophoblast cells (22, 23), leading to complete inhibition of VEGF signaling (lack of free VEGF; increase in VEGF-sFlt1 bound form), and ultimately resulting in the failure of differentiation of macrophages to the M2 phenotype and accumulation of large numbers of M1 cells in the placental bed.

Synthesizing these results, we therefore propose the following model for the role of VEGF in regulation of macrophage function in the establishment and maintenance of pregnancy (Fig 6). The initial highly inflammatory process associated with implantation leads to differentiation of primarily M1 macrophages. Later, large quantities of VEGF produced by decidual cells at the maternal-fetal interface not only stimulate recruitment of these cells to the site of implantation but also induce differentiation of these cells to the M2 phenotype. In preeclampsia, sFlt1 and possibly other factors produced by placental and decidual cells, act to disrupt this pathway and cause an accumulation of M1 macrophages. It remains unclear whether the shift in macrophage polarity seen in preeclampsia is a causative mechanism or a result of the disease itself. Additional studies in this area with decidual tissue samples from early pregnancies of women who later developed preeclampsia will be critical to gain a better understanding of the role of macrophage polarity in normal and pathologic pregnancies. In conclusion, although inhibition of VEGF signaling is known to be associated with preeclampsia, we demonstrate a role for VEGF in the regulation of macrophage function in the decidua and show a potential link between inhibition of VEGF signaling and the development of preeclampsia through dysregulation of macrophage function.
